# Development and validation of a nomogram to predict the prognosis of patients with gastric cardia cancer

**DOI:** 10.1038/s41598-020-71146-z

**Published:** 2020-08-24

**Authors:** Xiuquan Shi, Lijun Xu, Bingwei Ma, Siben Wang

**Affiliations:** 1 Department of General Surgery, The People’s Hospital of Yingshang, Fuyang, 236200 Anhui Province People’s Republic of China; 2 Department of Oncology, The People’s Hospital of Yingshang, Fuyang, 236200 Anhui Province People’s Republic of China; 3 Department of Gastrointestinal Surgery, Shanghai Tenth People’s Hospital, Tongji University, Shanghai, 200072 People’s Republic of China; 4 Department of Thoracic Surgery, Huainan First People’s Hospital, No. 203 Huaibin Road, Tianjiaan, Huainan, 232000 Anhui Province People’s Republic of China

**Keywords:** Cancer, Biomarkers, Oncology

## Abstract

Our goal was to develop a prognostic nomogram to predict overall survival (OS) and cancer-specific survival (CSS) in patients with gastric cardia cancer (GCC). Patients diagnosed with GCC from 2004 to 2015 were screened from the surveillance, epidemiology, and end results (SEER) database. A nomogram was developed based on the variables associated with OS and CSS using multivariate Cox analysis regression models, which predicted 3- and 5-year OS and CSS. The predictive performance of the nomogram was evaluated using the consistency index (C-index), calibration curve and decision curve analysis (DCA), and the nomogram was calibrated for 3- and 5-year OS and CSS. A total of 7,332 GCC patients were identified and randomized into a training cohort (5,231, 70%) and a validation cohort (2,200, 30%). Multivariate Cox regression analysis showed that marital status, race, SEER stage, grade, T stage, N stage, M stage, tumor size, and surgery were independent risk factors for OS and CSS in GCC patients. Based on the multivariate Cox regression results, we constructed prognostic nomograms of OS and CSS. In the training cohort, the C-index for the OS nomogram was 0.714 (95% CI = 0.705–0.723), and the C-index for the CSS nomogram was 0.759 (95% CI = 0.746–0.772). In the validation cohort, the C-index for the OS nomogram was 0.734 (95% CI = 0.721–0.747), while the C-index for the CSS nomogram was 0.780 (95% CI = 0.759–0.801). Our nomogram has better prediction than the nomogram based on TNM stage. In addition, in the training and external validation cohorts, the calibration curves of the nomogram showed good consistency between the predicted and actual 3- and 5-year OS and CSS rates. The nomogram can effectively predict OS and CSS in GCC patients, which may help clinicians personalize prognostic assessments and clinical decisions.

## Introduction

Gastric cancer is the 6th most common cancer and the 3rd leading cause of tumor-related death worldwide^1^. It was reported that there were approximately 27,510 new cases of gastric cancer resulting in 11,140 deaths in the United States in 2019^1^. Anatomically, gastric cancer can be divided into gastric cardiac carcinoma (GCC) and non-cardia gastric cancer (NGCC). In recent decades, although the overall incidence of gastric cancer has declined worldwide^2^, the incidence of GCC has increased^3^. This may be due to the increased incidence of gastroesophageal reflux disease and obesity^4^. According to previous reports, there are significant differences in incidence and prognostic specificity between GCC and NGCC, indicating that they are different tumor entities^5–7^. GCC has a poor prognosis and is a serious threat to human health^8^.

At present, the prognosis of GCC is mainly predicted by the TNM staging system^9^. The TNM classification proposed by the American Joint Commission on Cancer (AJCC) is the most widely used staging system, and it is mainly based on tumor invasion (T), regional lymph node (N) and distant metastasis (M) to predict the survival of cancer patients^10,11^. However, the evaluation of cancer prognosis based on TNM stage alone has limitations and cannot fully evaluate clinicopathological factors, such as age, sex, race and other factors.

The nomogram is a statistics-based tool that calculates the probability of clinical events by considering the preweight value of each factor^12,13^. In recent years, nomograms have been widely used to predict the survival rate of various cancers^11,14,15^. The purpose of this study is to develop an effective prognostic nomogram to predict the overall survival (OS) and cancer-specific survival (CSS) of patients with GCC to help clinicians provide personalized treatment recommendations.

## Results

### Demographic and pathologic characteristics

**Figure 1:**
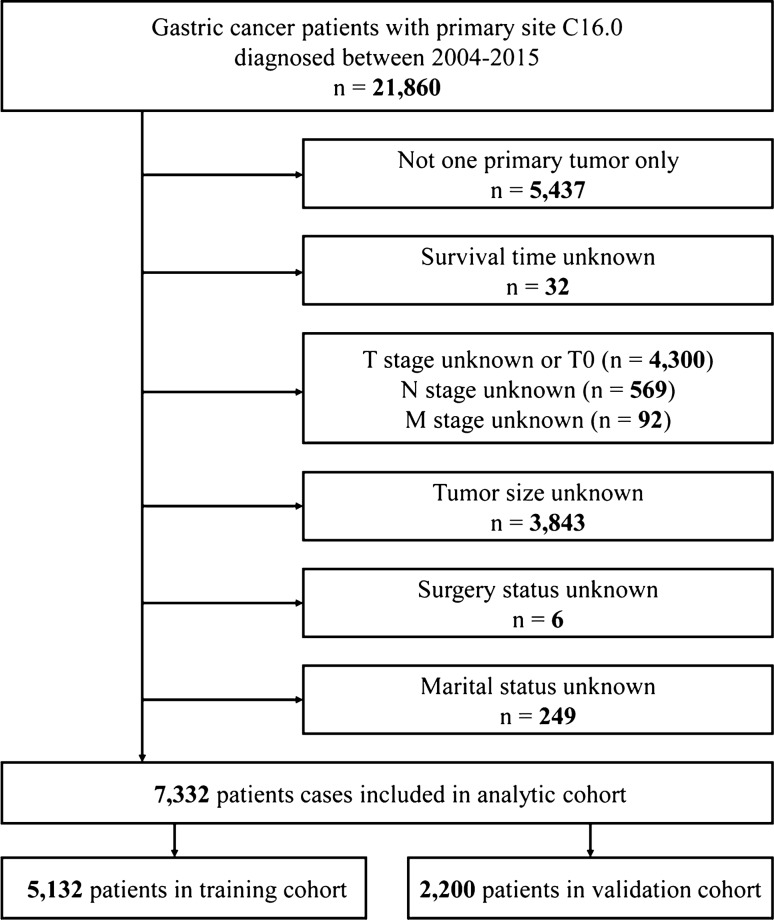
Schematic overview for patient identification.

A total of 7,332 patients were included in the study, and they were randomly assigned to two different cohorts: the training cohort (n = 5,132) and the validation cohort (n = 2,200). A flow chart showing the process of including patients in the study is depicted in Fig. 1. The demographic and pathologic characteristics of GCC patients are shown in Table 1. In our cohort, the highest incidence of GCC was in patients over 60 (64.7%) years old, and the majority of patients were male (79.6%), white (87.1%), and married (66.8%). The most common GCC classifications were adenocarcinoma (83.0%), regional (42.0%), grade III (51.4%) and M0 stage (78.3%). In addition, 62.8% of patients received surgery, and 64.9% received chemotherapy.

**Table 1:** Baseline demographic and clinical characteristics with gastric cardia cancer (GCC) patients in our study.

Characteristic	Total No. (%)	The training cohort	The validation cohort
		No. (%)	No. (%)
Total	7,332	5,132 (70.0)	2,200 (30.0)
**Sex**			
Male	5,836 (79.6)	4,088 (79.7)	1,748 (79.5)
Female	1,496 (20.4)	1,044 (20.3)	452 (20.5)
**Age at diagnosis**			
< 40	196 (2.7)	145 (2.8)	51 (2.3)
40–60	2,390 (32.6)	1649 (32.1)	741 (33.7)
> 60	4,746 (64.7)	3,338 (65.0)	1,408 (64.0)
**Marital status**			
Married	4,896 (66.8)	3,431 (66.9)	1,465 (66.6)
Divorced/separated	750 (10.2)	514 (10.0)	236 (10.7)
Widowed	721 (9.8)	505 (9.8)	216 (9.8)
Single	965 (13.2)	682 (13.3)	283 (12.9)
**Race**			
White	6,388 (87.1)	4,484 (87.4)	1,904 (86.5)
Black	385 (5.3)	255 (5.0)	130 (5.9)
Others	559 (7.6)	393 (7.7)	166 (7.5)
**Histological type**			
Adenocarcinoma	6,089 (83.0)	4,246 (82.7)	1,843 (83.8)
Others	1,243 (17.0)	886 (17.3)	357 (16.2)
**SEER stage**			
Localized	2,070 (28.2)	1,419 (27.7)	651 (29.6)
Regional	3,077 (42.0)	2,198 (42.8)	879 (40.0)
Distant	2,185 (29.8)	1515 (29.5)	670 (30.5)
**Grade**			
Grade I	395 (5.4)	283 (5.5)	112 (5.1)
Grade II	2,249 (30.7)	1,533 (29.9)	716 (32.5)
Grade III	3,767 (51.4)	2,678 (52.2)	1,089 (49.5)
Grade IV	155 (2.1)	112 (2.2)	43 (2.0)
Unknown	766 (10.4)	526 (10.2)	240 (10.9)
**T stage**			
T1	2,013 (27.5)	1,369 (26.7)	644 (29.3)
T2	3,215 (43.8)	2,261 (44.1)	954 (43.4)
T3	1,443 (19.7)	1,038 (20.2)	405 (18.4)
T4	661 (9.0)	464 (9.0)	197 (9.0)
**N stage**			
N0	2,863 (39.0)	1,984 (38.7)	879 (40.0)
N1	3,454 (47.1)	2,427 (47.3)	1,027 (46.7)
N2	773 (10.5)	550 (10.7)	223 (10.1)
N3	242 (3.3)	171 (3.3)	71 (3.2)
**M stage**			
M0	5,744 (78.3)	4,049 (78.9)	1,695 (77.0)
M1	1,588 (21.7)	1,083 (21.1)	505 (23.0)
**Tumor size**			
≤ 2 cm	1,581 (21.6)	1,100 (21.4)	481 (21.9)
2–5 cm	3,560 (48.6)	2,498 (48.7)	1,062 (48.3)
> 5 cm	2,191 (29.9)	1,534 (29.9)	657 (29.9)
**Surgery**			
Yes	4,602 (62.8)	3,224 (62.8)	1,378 (62.6)
No	2,730 (37.2)	1,908 (37.2)	822 (37.4)
**Chemotherapy**			
Yes	4,757 (64.9)	3,336 (65.0)	14,221 (64.6)
No/unknown	2,575 (35.1)	1796 (35.0)	779 (35.4)

Percentages may not total 100 because of rounding.

*SEER* surveillance, epidemiology, and end results.

### Identification of prognostic factors of OS and CSS

To identify the prognostic factors, we performed univariate and multivariate Cox regression analyses in the training cohort. According to the univariate Cox analysis, age at diagnosis, marital status, race, histological type, SEER stage, grade, T stage, N stage, M stage, tumor size, and surgery were significantly associated with OS, while sex, age, marital status, race, histological type, SEER stage, grade, T stage, N stage, M stage, tumor size, and surgery were closely related to CSS. These significant variables were further entered into the multivariate Cox analysis. Multivariate Cox analysis showed that age, marital status, race, SEER stage, grade, T stage, N stage, M stage, tumor size and surgery were independent prognostic factors for OS (Table 2). Regarding CSS, eleven variables, including sex, age, marital status, race, SEER stage, grade, T stage, N stage, M stage, tumor size and surgery, were identified as independent prognostic factors (Table 3).

**Table 2:** Univariate and multivariate analysis of overall survival (OS) rates in the training cohort.

	Univariate analysis		Multivariate analysis^a^	
	Hazard ratio (95% CI)	*P* value	Hazard ratio (95% CI)	*P* value
**Sex**				
Male	Reference			
Female	0.96 (0.89–1.04)	0.319		
**Age at diagnosis**				
< 40	Reference		Reference	
40–60	1.00 (0.81–1.22)	0.748	1.06 (0.86–1.30)	0.603
> 60	1.26 (1.03–1.54)	0.024	1.47 (1.20–1.80)	< 0.001
**Marital status**				
Married	Reference		Reference	
Divorced/separated	1.17 (1.05–1.30)	0.005	1.20 (1.08–1.34)	0.001
Widowed	1.50 (1.35–1.67)	< 0.001	1.34 (1.20–1.49)	< 0.001
Single	1.26 (1.14–1.38)	< 0.001	1.18 (1.07–1.30)	0.001
**Race**				
White	Reference		Reference	
Black	1.37 (1.19–1.58)	< 0.001	1.19 (1.04–1.38)	0.015
Others	0.90 (0.80–1.02)	0.107	0.90 (0.80–1.02)	0.109
**Histological type**				
Adenocarcinoma	Reference		Reference	
Others	1.33 (1.22–1.44)	< 0.001	–	0.193
**SEER stage**				
Regional	Reference		Reference	
Localized	1.57 (1.53–1.63)	< 0.001	1.65 (1.57–1.74)	< 0.001
Distant	1.97 (1.84–2.13)	< 0.001	2.95 (2.84–3.07)	< 0.001
**Grade**				
Grade I	Reference		Reference	
Grade II	1.43 (1.20–1.70)	< 0.001	1.21 (1.01–1.43)	0.036
Grade III	2.09 (1.77–2.47)	< 0.001	1.53 (1.29–1.81)	< 0.001
Grade IV	1.95 (1.49–2.55)	< 0.001	1.43 (1.09–1.87)	0.010
Unknown	1.91 (1.57–2.31)	< 0.001	1.18 (0.97–1.44)	0.090
**T stage**				
T1	Reference		Reference	
T2	1.21 (1.12–1.32)	< 0.001	0.98 (0.89–1.07)	0.654
T3	1.31 (1.19–1.44)	< 0.001	1.06 (0.95–1.18)	0.314
T4	2.48 (2.21–2.80)	< 0.001	1.28 (1.13–1.46)	< 0.001
**N stage**				
N0	Reference		Reference	
N1	1.52 (1.41–1.64)	< 0.001	0.97 (0.87–1.08)	0.534
N2	1.77 (1.59–1.97)	< 0.001	1.27 (1.10–1.45)	0.001
N3	2.27 (1.91–2.69)	< 0.001	1.38 (1.14–1.67)	0.001
**M stage**				
M0	Reference		Reference	
M1	3.09 (2.87–3.33)	< 0.001	1.68 (1.47–1.92)	< 0.001
**Tumor size**				
≤ 2 cm	Reference		Reference	
2–5 cm	1.93 (1.76–2.12)	< 0.001	1.39 (1.26–1.53)	< 0.001
> 5 cm	2.17 (1.97–2.40)	< 0.001	1.37 (1.23–1.53)	< 0.001
**Surgery**				
Yes	Reference		Reference	
No	3.07 (2.87–3.28)	< 0.001	2.43 (2.24–2.63)	< 0.001
**Chemotherapy**				
Yes	Reference			
No/unknown	0.95 (0.89–1.02)	0.149		

*OS* overall survival, *SEER* surveillance, epidemiology, and end results.

^a^Model was adjusted by age at diagnosis, marital status, race, histological type, SEER stage, grade, TNM stage, tumor size and surgery.

**Table 3:** Univariate and multivariate analysis of cancer-specific survival (CSS) rates in the training cohort.

	Univariate analysis		Multivariate analysis^a^	
	Hazard ratio (95% CI)	*P* value	Hazard ratio (95% CI)	*P* value
**Sex**				
Male	Reference		Reference	
Female	1.38 (1.22–1.57)	< 0.001	1.33 (1.16–1.52)	< 0.001
**Age at diagnosis**				
< 40	Reference		Reference	
40–60	0.70 (0.52–0.95)	0.023	0.80 (0.59–1.09)	0.159
> 60	0.89 (0.66–1.20)	0.459	1.18 (0.87–1.60)	0.292
**Marital status**				
Married	Reference		Reference	
Divorced/separated	0.99 (0.81–1.21)	0.942	0.99 (0.81–1.21)	0.929
Widowed	1.71 (1.45–2.03)	< 0.001	1.38 (1.15–1.66)	0.001
Single	1.34 (1.14–1.57)	< 0.001	1.19 (1.01–1.41)	0.039
**Race**				
White	Reference		Reference	
Black	2.03 (1.65–2.50)	< 0.001	1.75 (1.42–2.17)	< 0.001
Others	1.58 (1.33–1.88)	< 0.001	1.54 (1.30–1.84)	< 0.001
**Histological type**				
Adenocarcinoma	Reference		Reference	
Others	1.61 (1.41–1.84)	< 0.001	-	0.111
**SEER stage**				
Regional	Reference		Reference	
Localized	1.48 (1.40–1.56)	< 0.001	1.57 (1.45–1.71)	< 0.001
Distant	2.46 (2.18–2.78)	< 0.001	2.87 (2.69–3.08)	< 0.001
**Grade**				
Grade I	Reference		Reference	
Grade II	1.66 (1.17–2.36)	0.005	1.32 (0.93–1.89)	0.119
Grade III	3.13 (2.23–4.39)	< 0.001	2.05 (1.45–2.89)	< 0.001
Grade IV	3.02 (1.87–4.88)	< 0.001	1.95 (1.20–3.15)	0.007
Unknown	2.66 (1.83–3.87)	< 0.001	1.44 (0.99–2.10)	0.060
**T stage**				
T1	Reference		Reference	
T2	0.98 (0.85–1.13)	0.807	0.70 (0.60–0.82)	< 0.001
T3	1.05 (0.89–1.25)	0.550	0.75 (0.62–0.91)	0.003
T4	2.95 (2.47–3.54)	< 0.001	1.19 (0.98–1.45)	0.074
**N stage**				
N0	Reference		Reference	
N1	1.56 (1.37–1.78)	< 0.001	0.99 (0.83–1.18)	0.916
N2	2.33 (1.96–2.77)	< 0.001	1.64 (1.32–2.04)	< 0.001
N3	2.84 (2.17–3.72)	< 0.001	1.59 (1.18–2.14)	0.002
**M stage**				
M0	Reference		Reference	
M1	4.32 (3.84–4.87)	< 0.001	2.19 (1.74–2.75)	< 0.001
**Tumor size**				
≤ 2 cm	Reference		Reference	
2–5 cm	2.56 (2.13–3.08)	< 0.001	1.75 (1.43–2.13)	< 0.001
> 5 cm	3.59 (2.97–4.34)	< 0.001	2.08 (1.69–2.57)	< 0.001
**Surgery**				
Yes	Reference		Reference	
No	3.56 (3.18–3.99)	< 0.001	2.45 (2.13–2.81)	< 0.001
**Chemotherapy**				
Yes	Reference			
No/unknown	0.97 (0.86–1.10)	0.648		

*CSS* cancer-specific survival, *SEER* surveillance, epidemiology, and end results.

^a^Model was adjusted by sex, age at diagnosis, marital status, race, histological type, SEER stage, grade, TNM stage, tumor size and surgery.

### Nomograms construction and performance assessment

**Figure 2:**
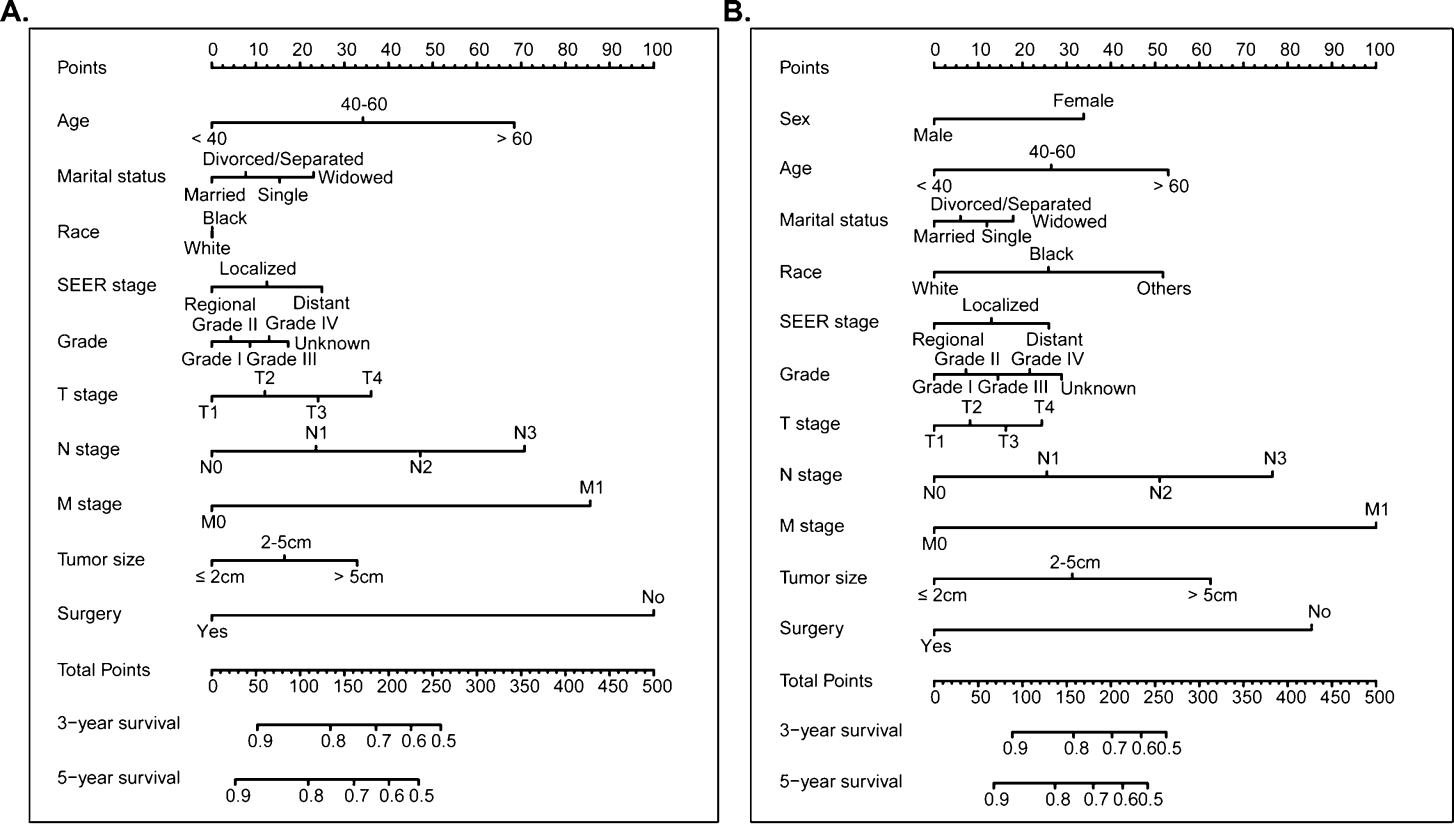
Nomogram predicting 3-, and 5-year overall survival (OS) and cancer-specific survival (CSS) rate of GCC patients. (**A**) OS rate; (**B**) CSS rate.

We developed two nomograms for OS and CSS: one was based on the results of multivariate Cox analysis (Fig. 2), and the other was based on TNM stage (Supplementary Fig. S1). Each of the variables was given a point according to the HR. Then, by adding the total score of each variable and locating the score on the total points scale, the probability of 3- and 5-year OS and CSS can be obtained. In the nomogram of OS, surgery contributed the greatest to the survival outcome, while M stage contributed the greatest in the nomogram of CSS.

**Figure 3:**
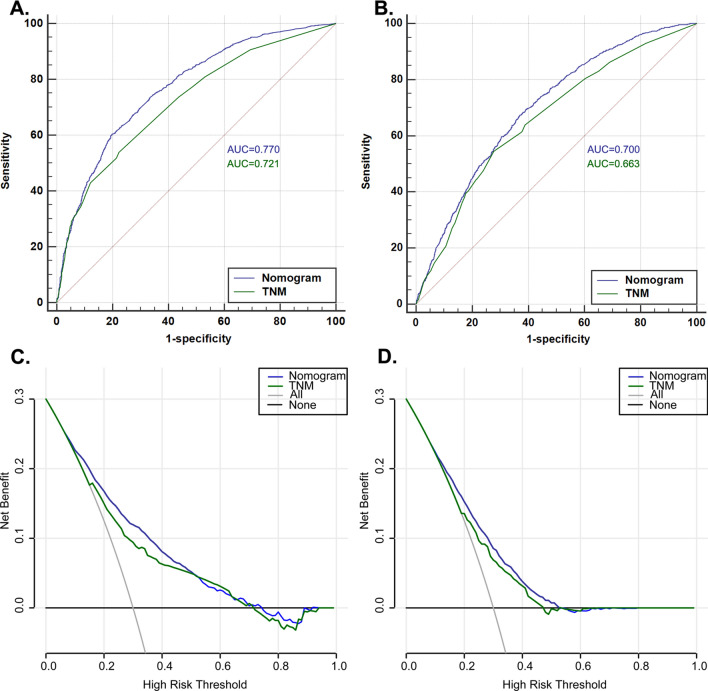
Receiver operating characteristic (ROC) and decision curve analysis (DCA) curves detect the predictive value of two nomograms in GCC prognosis. (**A**) ROC curve for overall survival (OS); (**B**) ROC for cancer-specific survival (CSS); (**C**) DCA for overall survival (OS); (**D**) DCA for cancer-specific survival (CSS).

Analysis of the time-dependent ROC curves for OS and CSS showed that the AUCs of the nomograms (OS: 0.770, 95% CI = 0.758–0.782; CSS: 0.700, 95% CI = 0.687–0.713) were significantly larger than those of TNM stage (OS: 0.721, 95% CI = 0.709–0.734; CSS: 0.663, 95% CI = 0.650–0.676) in the training cohort (Table 4 and Fig. 3A, B). To compare whether the predicted survival time was consistent with the actual survival time, the C-index was used to verify the nomogram in the training cohort. For OS or CSS, the C-index of the nomograms (OS, C-index = 0.714; CSS, C-index = 0.759) was greater than that of the TNM stage (OS, C-index = 0.651; CSS, C-index = 0.7696). Similar results were found in the validation cohort (Table 5). This similarity of the results indicates that the model established by the nomogram was accurate.

**Table 4:** Comparison of area under the curve (AUC) between the nomogram and TNM stage in gastric cardia cancer (GCC) patients**.**

Characteristics	Training cohort			Validation cohort		
	AUC	95% CI	*P* value	AUC	95% CI	*P* value
**OS**						
Nomogram	0.770	0.758–0.782		0.784	0.766–0.801	
TNM stage	0.721	0.709–0.734	< 0.001	0.706	0.686–0.725	< 0.001
**CSS**						
Nomogram	0.700	0.687–0.713		0.706	0.687–0.725	
TNM stage	0.663	0.650–0.676	< 0.001	0.679	0.659–0.698	0.009

*AUC* area under the curve, *CI* confidence interval.

**Table 5:** Comparison of C-indexes between the nomogram and TNM stage in gastric cardia cancer (GCC) patients**.**

Characteristics	Training cohort			Validation cohort		
	HR	95% CI	*P* value	HR	95% CI	*P* value
**OS**						
Nomogram	0.714	0.705–0.723		0.734	0.721–0.747	
TNM stage	0.651	0.641–0.661	< 0.001	0.653	0.638–0.668	< 0.001
**CSS**						
Nomogram	0.759	0.746–0.772		0.780	0.759–0.801	
TNM stage	0.696	0.679–0.713	< 0.001	0.619	0.594–0.644	< 0.001

*HR* hazard ratio, *CI* confidence interval.

**Figure 4:**
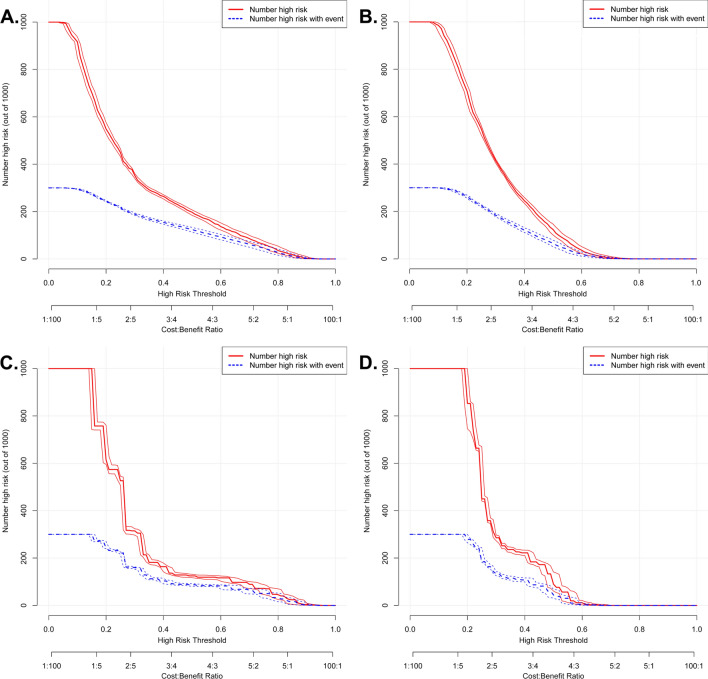
Clinical impact curve (CIC) detects the predictive value of two nomograms in GCC prognosis. (**A**,**B**) All variables nomogram. (**C**,**D**) TNM stage nomogram.

**Figure 5:**
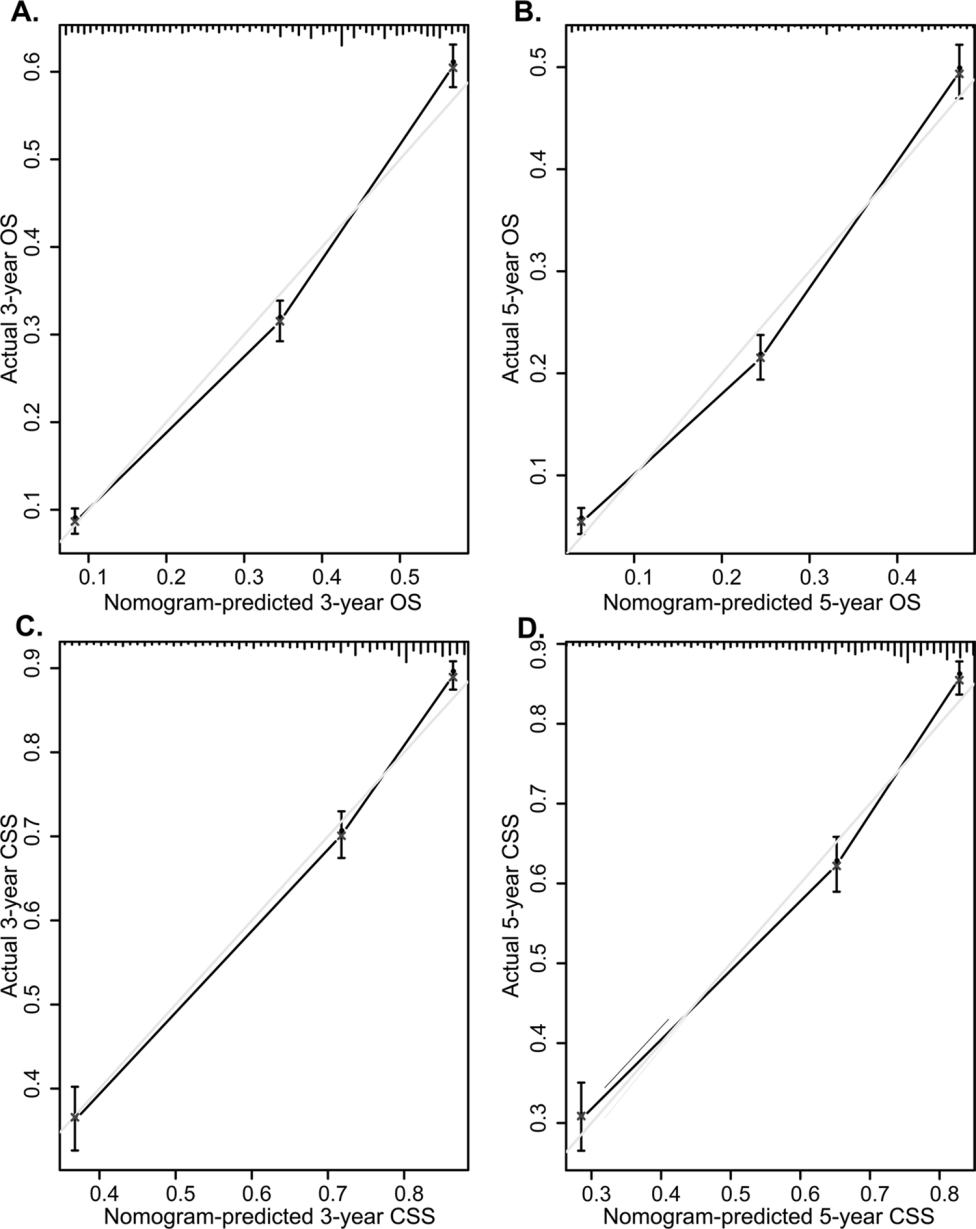
Calibration plot of the nomogram for predicting 3-, 5-, and 10‐year overall survival (OS) and cancer-specific survival (CSS) in the training cohort. (**A**) 3-year OS; (**B**) 5-year OS; (**C**) 3-year CSS; (**D**) 5-year CSS.

In addition, DCA calculates the net benefit to evaluate the clinical utility of the nomogram. The results showed that in the broad threshold of OS (10–50%), the clinical net benefit of the nomograms was greater than that of the TNM stage (Fig. 3C,D). The CIC results show that among the broad thresholds for OS (20–70%), the nomograms were classified as positive, and the number of true positives was greater than those of the TNM stage (Fig. 4). Moreover, we calibrated the 3- and 5-year OS and CSS nomograms of the training cohort (Fig. 5) and the validation cohort (Supplementary Fig. S2), which were very close to the ideal curve. This showed good consistency between the prediction of the nomogram and the actual observed outcomes in the training and validation cohorts.

## Discussion

GCC is a kind of malignant tumor at the junction of the stomach and esophagus that mostly occurs in middle-aged people over 40 years old and elderly people and that accounts for approximately 10% of all digestive system tumors^16,17^. After the onset of the disease, patients often have clinical symptoms such as upper gastrointestinal bleeding, dysphagia, and stomach discomfort. The prognosis of patients with GCC is poor^18^, so it is very important to develop an effective system to predict the prognosis of these patients.

In this study, we first developed prognostic nomograms of OS and CSS in patients with GCC. We used the SEER database to conduct Cox regression analysis on many GCC patients to identify independent risk factors for OS and CSS. We constructed two prognostic nomograms: one based on multivariate Cox analysis and the other based on TNM stage. Through the examination of the C-index, ROC curve, DCA curve and CIC, it was found that our nomogram had better prognostic ability than that of the TNM stage. In addition, we verified and calibrated the nomogram and evaluated the accuracy of OS and CSS nomograms for 3 and 5 years. The results show that the predicted results of the nomogram are in good agreement with the actual observed results and are supported by the calibration curve, ROC curve analysis and C-index value. The C-index of the nomograms is more than 0.7, indicating that it has sufficient discrimination ability. The DCA results show that the nomogram we developed has good clinical practical value.

Recently, some nomograms containing various input variables have been developed to predict the prognosis of different digestive tract tumors^19–22^. Kim et al.^19^ developed and validated a nomogram that predicted the risk of lymph node metastasis in patients with early gastric cancer and could be used to avoid unnecessary gastrectomy after endoscopic dissection. By analyzing 9,026 patients with metastatic esophageal cancer between 2010 and 2015, Zhu et al.^20^ found that the nomogram was better at predicting distant metastasis of esophageal cancer than traditional TNM staging. Similarly, the nomogram developed by Xue et al.^21^ that includes nutritional and immune parameters can effectively predict the overall and postoperative survival rate and relapse-free survival of gastric cancer patients after radical gastrectomy, and its prediction accuracy and discrimination ability are better than those of TNM staging.

In the current reports on GCC, it is remarkable that Liu et al.^22^ developed a nomogram for predicting the total survival rate of GCC based on radiology and clinical predictors. However, the nomogram for predicting the OS of GCC based on radiology and clinical predictors is not available, and the nomogram for CSS is applicable for a limited population, since it is only suitable for patients undergoing preoperative radiotherapy.

TNM staging was determined according to the results of laboratory examination and postoperative pathological examination. For example, Gong et al.^23^ found that TNM stage was associated with the prognosis of high gastric cancer, and T stage was an independent factor for lymph node metastasis. Zhu et al.^24^ found that TNM staging was associated with patients with adenocarcinoma of the esophagogastric junction, but only N staging was an independent risk factor for prognosis. TNM staging is a common method to predict the prognosis of GCC patients, but TNM staging has limitations and cannot provide clinicians with personalized prognosis prediction. In this study, we successfully constructed a practical nomogram based on thirteen factors: sex, age, marital status, race, histological type, SEER stage, grade, T stage, N stage, M stage, tumor size, surgery, and chemotherapy, and its prediction power was better than that of traditional TNM staging.

Our research still has some limitations. First, the lack of treatment information (chemotherapy regimens, surgical methods, etc.) in the SEER database may alter our results. Second, our study is a retrospective study with inevitable selection bias. Third, due to the lack of external verification, we are concerned about the generality of our model and may need further research to prove our results.

## Conclusions

The present study demonstrated that the nomogram is a better prognostic determinant than TNM staging systems in GCC patients. The nomogram we developed accurately and reliably predicted the 3- and 5-year OS and CSS of GCC. This model could enable clinicians to more precisely estimate the survival of GCC patients.

## Patients and methods

### Patient selection

The SEER database is an open public database and provides cancer data (e.g., treatment, primary site, tumor size, tumor stage, treatment regimen, pathological type, time of death, and cause of death) from the population based registries of 18 sites that cover approximately 28% of the USA population^25^. The National Cancer Institute's SEER*Stat software version 8.3.6 (https://seer.cancer.gov/seerstat/) (SEER 18 Regs Custom Data (with additional treatment fields), Nov 2018 Sub (2004–2016 varying) database) was used in this study.

The International Classification of Diseases for Oncology (ICD-O) site code C16.0 was used to identify patients diagnosed with GCC between 2004 and 2015. In total, 21,860 patients were enrolled in this study according to the specified inclusion and exclusion criteria. The exclusion criteria in our study were as follows: more than one primary tumor (n = 5,437); unknown survival time (n = 32); unknown T stage and T0 (n = 4,300); unknown N stage (n = 569); unknown M stage (n = 92); unknown tumor size (n = 3,843); unknown surgery status (n = 6); and unknown marital status (n = 249). In total, 7,332 GCC patients were included for this analysis.

Data regarding sex, age, marital status, race, histological type, SEER stage, grade, T stage, N stage, M stage, tumor size, surgery, chemotherapy, vital status, and survival time were extracted from the SEER database (2004–2015) for further analysis. OS duration was defined as the time from diagnosis until death or last follow-up. CSS duration was defined as the time from diagnosis until death because of GCC or last follow-up.

### Statistical analyses

Cases were randomly divided into training and validation cohorts (ratio 7:3). Univariate and multivariate Cox regression models were applied to calculate the hazard ratio (HR) and 95% confidence interval (CI) to assess the independent contributions of each factor to OS and CSS. In the univariate Cox proportional hazard model, variables with *P* < 0.05 were further analyzed in the multivariate Cox proportional hazard model. Based on multivariate Cox analysis, a nomogram was developed to predict the 3- and 5-year OS and CSS rates. In contrast, we built another nomogram based on TNM stage. Then, we used MedCalc software (version 15.2.0) to generate the receiver operating characteristic (ROC) curve for the two nomograms and determined the area under the curve (AUC). The performance of the nomogram was assessed by the C-index and the calibration curve (1,000 bootstrap resamples). The C-index has a range from 0.5 to 1.0, with 0.5 indicating random chance and 1.0 considered perfect discrimination. Decision curve analysis (DCA) and a clinical impact curve (CIC) were employed to evaluate the net benefit of the nomogram in a clinical context.

The above statistical analyses were conducted using SPSS software version 24.0 (SPSS, Chicago, USA) and the statistical software package R version 3.5.3 (https://www.r-project.org/). All tests were two-sided. A *P *value ≤ 0.05 (two-sided) was considered statistically significant.

### Research involving human participants and/or animals

This article does not contain any studies with human participants or animals performed by any of the authors.

## Supplementary information

Supplementary information.
