# Supplementary material for: Development and validation of a nomogram to predict the prognosis of patients with gastric cardia cancer
Source: Sci Rep. 2020 Aug 24;10:14143. doi: 10.1038/s41598-020-71146-z (PMC7445298; doi:10.1038/s41598-020-71146-z)
Supplement: Supplementary file 1 — Supplementary information. [file 41598_2020_71146_MOESM1_ESM.docx]

**Development and validation of a** **nomogram to predict the prognosis of patients with gastric cardia cancer**

Xiuquan Shi^1 * #^, Lijun Xu^2 *^, Bingwei Ma^3 *^, Siben Wang^4 #^

**
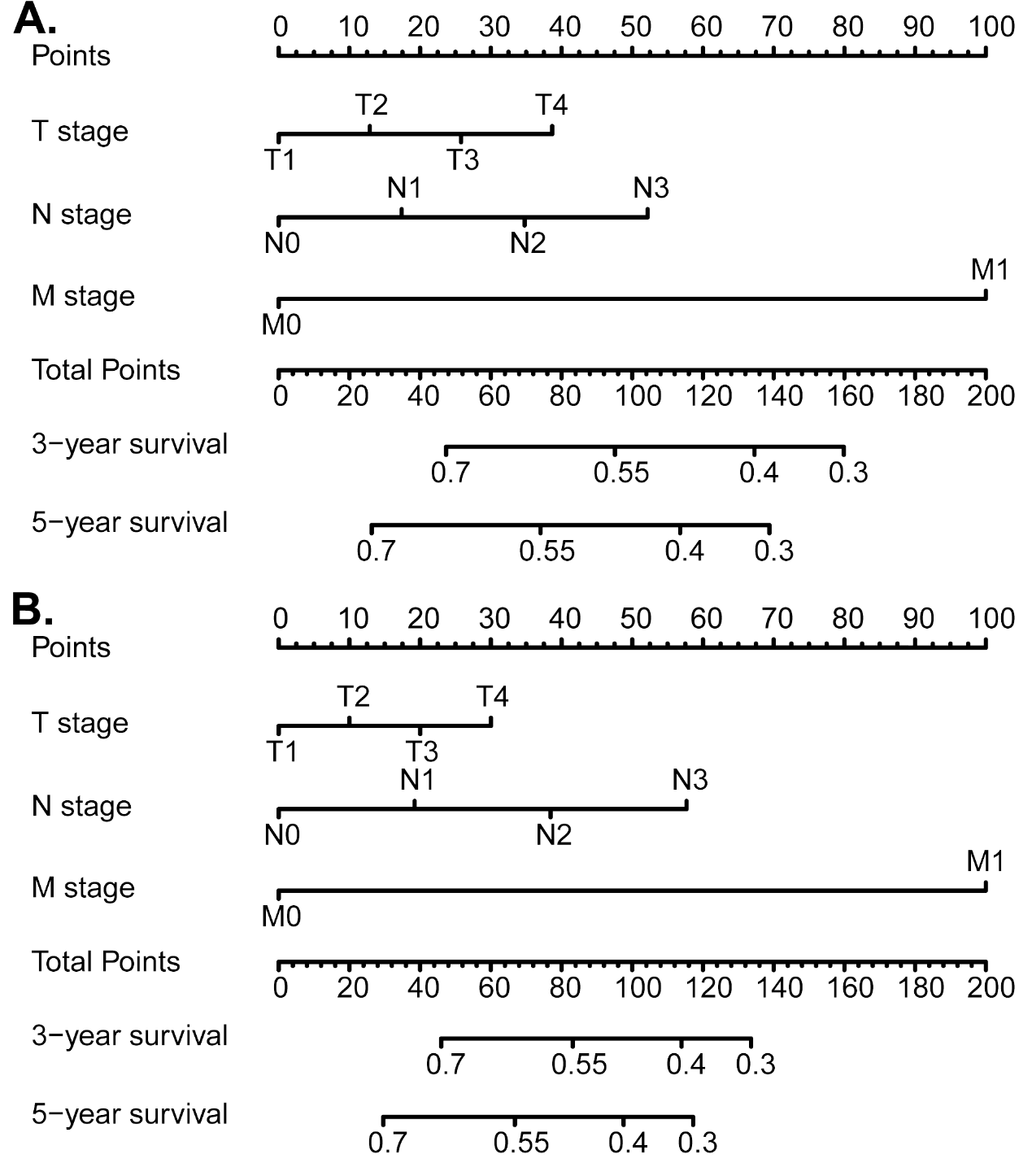
**

**Figure S1.** TNM stage predicting 3-, and 5-year overall survival (OS) and cancer-specific survival (CSS) rate of GCC patients. A, OS rate; B, CSS rate.

**
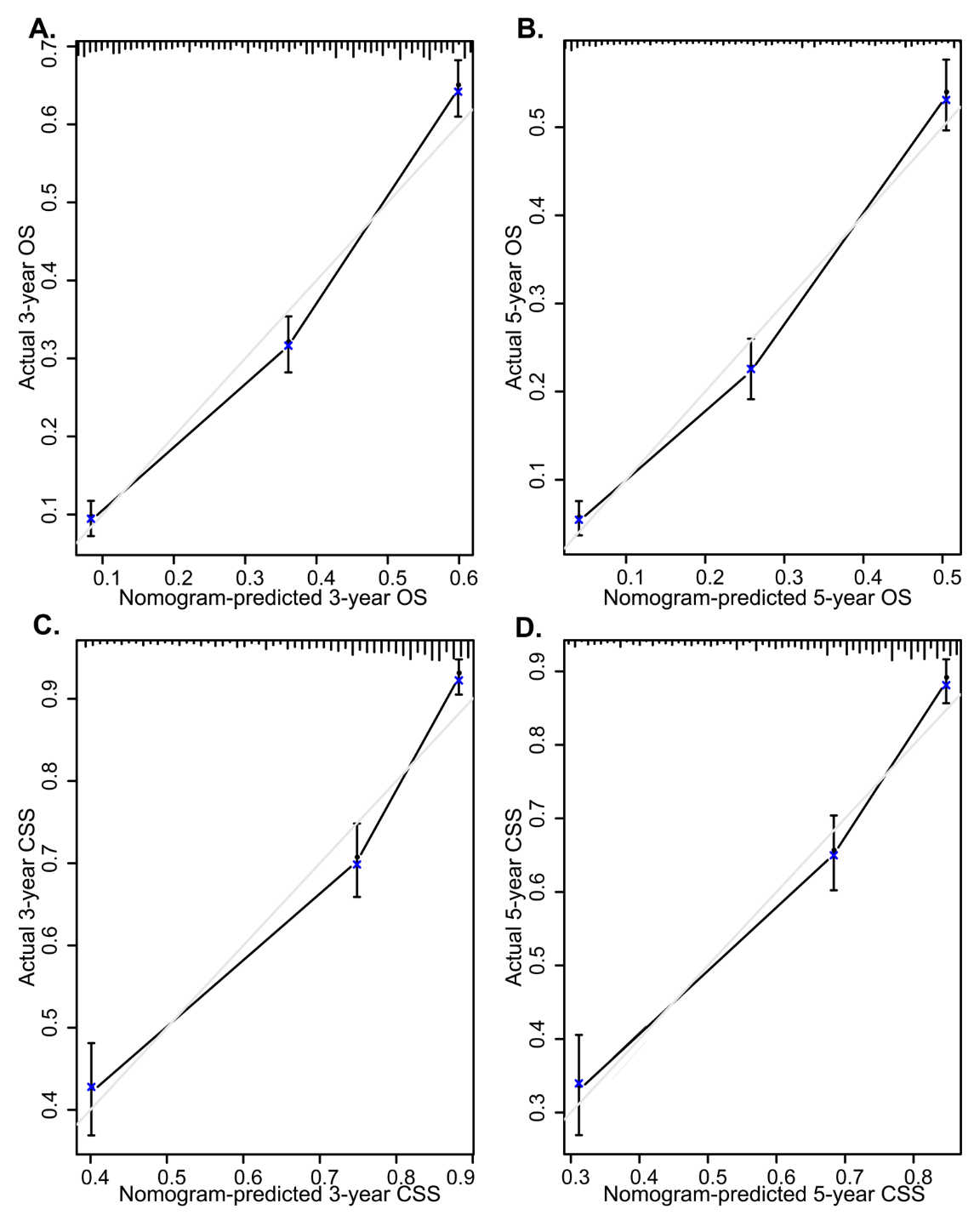
**

**Figure S2.** Calibration plot of the nomogram for predicting 3- and 5-year overall survival (OS) and cancer-specific survival (CSS) in the validation cohort. A. 3-year OS; B. 5-year OS; C. 3-year CSS; D. 5-year CSS.
